# Oncologic results of Nephron sparing endoscopic approach for upper tract low grade transitional cell carcinoma in comparison to nephroureterectomy – a case control study

**DOI:** 10.1186/1471-2490-14-97

**Published:** 2014-12-02

**Authors:** Azik Hoffman, Ofer Yossepowitch, Yaron Erlich, Ronen Holland, David Lifshitz

**Affiliations:** Institute of Urology, Rabin Medical Center - Beilinson Campus, Petah Tikva, and Sackler Faculty of Medicine, Tel Aviv University, Petah Tikva, 49100 Tel Aviv, Israel

**Keywords:** Upper tract TCC, Nephron sparing, Ureteroscopy

## Abstract

**Background:**

There is paucity of data as to the results of the endoscopic approach in comparison to the golden standard of nephro-ureterectomy in elective, low grade TCC, patients. Our purpose is to report our results of a nephron sparing approach compared to nephro-ureterectomy in those patients.

**Methods:**

From a retrospective data base we identified 25 patients and 23 patients who underwent a nephron sparing ureterosocpic resection and nephro-reterectomy for low grade UT-TCC, respectively. The endoscopic technique included endoscopic tumor biopsy followed by primary resection and/or fulguration. The nephron sparing group was followed by bi-annual ureteroscopy and upper tract imaging, timely cystoscopy and urine cytology collection. Data for overall and disease related mortality, bladder and ureteral TCC recurrence and renal function are reported in both groups.

**Results:**

Median follow - up time was 26 months. 11 (44%) patients developed bladder recurrence at a median period of 9 months after initial ureteroscopy, compared to 9 (39%) in the NUx group (P < 0.05). Recurrent ureteral low grade TCC was observed in 9 patients (median: 9 months). All were treated endoscopicaly successfully. Renal function remained stable in the nephron sparing group. No disease related mortality was recorded in the nephron-sparing group while one patient died of his disease following NUx.

**Conclusions:**

Disease related mortality following a nephron sparing endoscopic approach or nephroureterectomy for low grade upper tract TCC is excellent. However, the nephron sparing approach is associated with a relatively high rate of ureteral and bladder recurrence. Therefore, a stringent follow-up protocol is required.

## Background

The nephron-sparing approach for upper tract TCC was traditionally reserved for patients with imperative indications such as solitary kidney, renal insufficiency or significant co-morbidities [1, 2]. With the advent of modern endoscopic techniques the indications for a nephron sparing endoscopic approach expanded. In the recent EAU guidelines on urothelial carcinoma of the upper urinary tract elective conservative management is indicated for unifocal, small, low grade, non-infiltrative tumors [3]. When choosing this approach, the main concern is tumor progression or up-grading. However, only patients with primary high-grade and non-Ta stage tumors were found to be at significant risk of death from UT-TCC [3]. The concept of a nephron sparing approach was studied widely in patients with RCC. Studies have shown that decreased renal function is associated with significant morbidity [4]. Therefore, partial nephrectomy is now the new standard of care when indicated. The same concept is gaining acceptance in UT-TCC. However, there is paucity of data comparing the oncological outcome of the nephron sparing approach in comparison to the gold standard of radical nephro-ureterectomy with excision of bladder cuff. In the current study we have conducted a retrospective comparison of the midterm follow-up results in patients with low grade UT-TCC treated by nephron-ureterectomy or an endoscopic resection.

## Methods

A systemic review for nephron-sparing management for patient with upper tract TCC was collected out of an institutional database including over 2700 ureteroscopic surgery performed between the years 2000–2010 in our institute. Patient with high grade upper tract TCC were excluded. Over 150 nephro-ureterectomy surgery were performed in the same period of time, 22 of them for upper tract low grade TCC.

### Patients

25 (0.9%) patients were identified in our database with low grade UT-TCC that was managed by endoscopic approach (group 1), compared to 22 patients who had nephro-ureterectomy due to endoscopically un-resectable (group 2) low grade UT-TCC. All patients in group 1 had tumors ≤1.5 cm that had papillary ureteroscopic appearance and could be completely resected.

### Endoscopic surgical technique

Renal and proximal ureter tumors were resected and ablated using a combination of holmium laser energy and monopolar electrocautry with a 1.9 FR bugbee electrode. For distal ureter tumors a 10.5 Fr. ureteroresectoscope (storz^©^) was preferentially utilized. A pathological specimen was obtained prior to resection during ureteroscopy.

Nephroureterectomy was performed open or laparoscopic with open distal ureter release and formal bladder cuff excision and bladder two-layer closure.

### Follow-up protocol

After primary resection, all patients were submitted to a pre-planned cystoscopy and urine cytology every 3 months for three years. Ureteroscopy was performed 3 months after initial resection and every 6 months thereafter if no recurrence was noted. CT urography or intravenous pyelography was performed every 6 months alternating with follow-up ureteroscopy. Kidney GFR was assessed using MDRD calculation once a year [5]. All participants have expressed their Informed consent, and the study was approved by the local (Rabin Medical Center) ethics (Helsinki) committee.

### Statistical analysis

Age and creatinine clearance were compared using student's *T*-test. Bladder cancer free probability was analyzed using log rank analysis.

## Results

**Figure 1:**
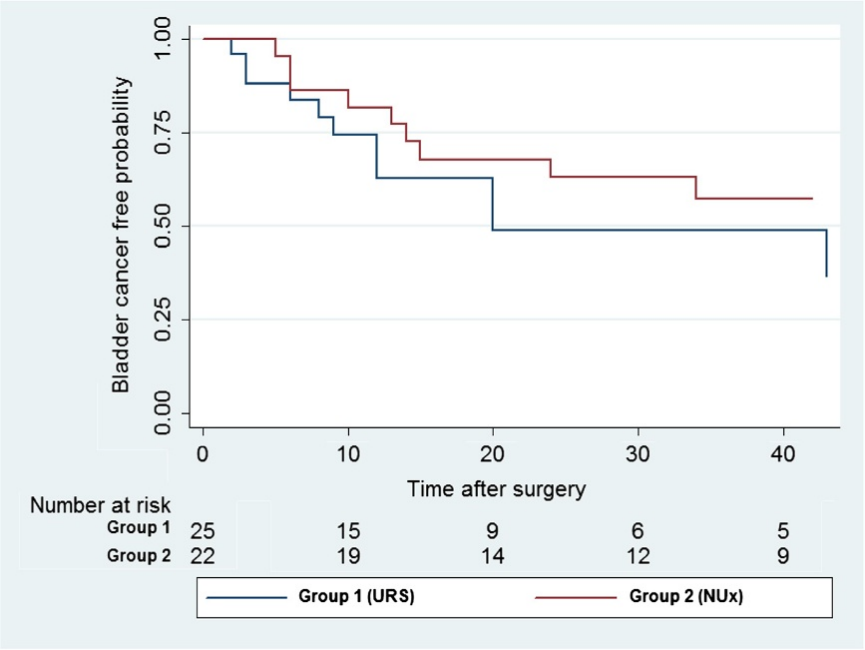
**Bladder recurrence.**

Median patient age at diagnosis was 64 years (range: 42–85) and 76 years (50–89) in the endoscopic resection group (group 1) and NUx group (group 2), respectively (p < 0.05). Primary diagnostic ureteroscopy identified Ta low grade TCC in 21 patients and non-conclusive result (due to lack of proper biopsy tissue) in four patients in group 1. Median follow up time was 26 months (range: 12–126) and 57 months (range: 12–149), respectively (Table 1). Patient data and primary location of the upper tract tumor is also shown in Table 1. Median follow time up after primary resection was 26 months (range: 12–126) and 57 months (range: 12–149) in groups 1 and 2, respectively.Bladder TCC free probability is illustrated in Figure 1.

**Table 1:** **Group data**

	Endoscopic resection group (N = 25)	Nephroureterectomy group (N = 22)	P value
Median age at diagnosis, months (range),	64 (42–85)	76 (50–89)	< 0.05
Median follow up, months (range)	26 (12–126)	57 (12–149)	
Primary tumor location (%)			
Kidney + renal pelvis	6 (24%)	10 (45%)	
Proximal ureter	3 (12%)	5 (23%)	
Distal ureter	17 (68%)	8 (36%)	
Pre OP creatinine clearance (MDRD)	66	68	>0.05
Post OP creatinine clearance (MDRD)	62	58	>0.05

**Figure 2:**
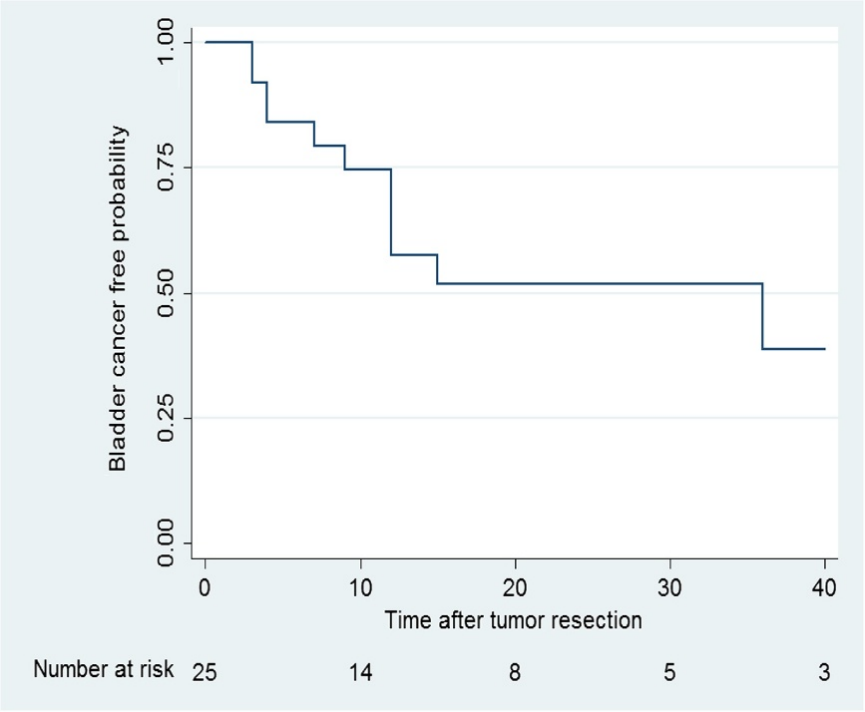
**Ureteral recurrence.**

In group 1, 11 patients (44%) developed bladder recurrence at a median period of 9 months (range: 2–43). Four (16%) patients had more than one recurrence, compared to 9 (39%) patients in group 2 (median time 13 months, range: 5–34). Three bladder recurrences were detected by positive cytology during follow up and Four (16%) had more than one bladder recurrence in group 1. All bladder recurrences were non-muscle invasive and were managed successfully by trans-urethral complete resection.Ureteral TCC free probability is illustrated in Figure 2.

Recurrent ureteral low grade TCC was observed in 9 patients (median: 9 months, range: 3–36) in group 1. All were re-treated successfully by a second endoscopic procedure. No case of pathological ureteral TCC up-grading or up-staging was observed during follow-up period.

**Figure 3:**
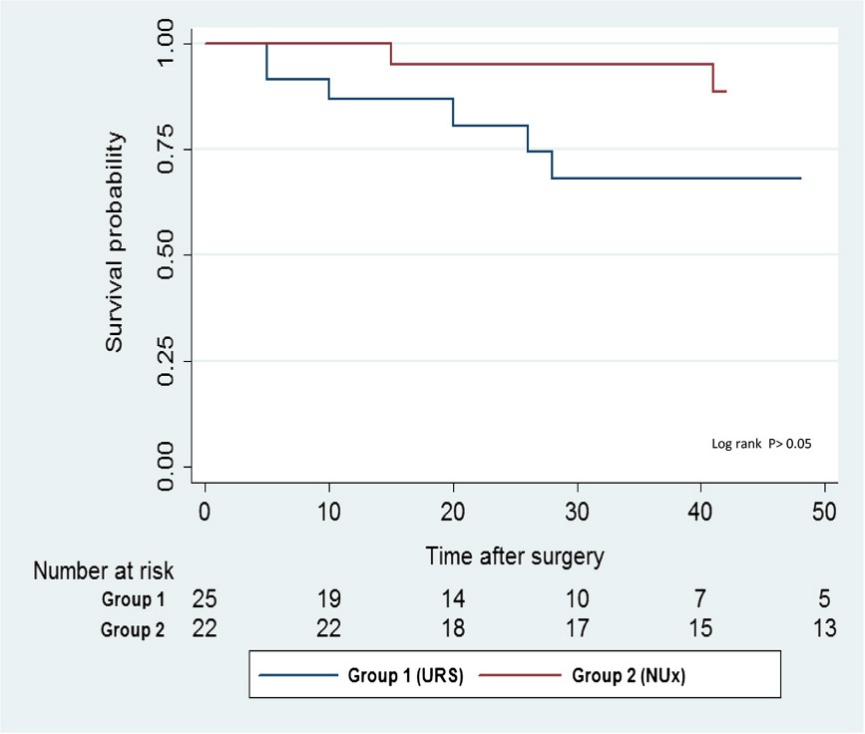
**Overall survival.**

Renal function, as calculated according to MDRD formula, remained stable in all patients in group 1 during follow-up, including six patients who presented with stage 3 chronic kidney disease (median eGFR 53 ml/min/1.73 m^2^) (Table 1).Overall survival rates are illustrated in Figure 3.

No disease related mortality was observed in group 1. In group 2, one patient died of muscle invasive bladder TCC ten years after initial UT-TCC diagnosis. Seven patients (28%) in group 1 died of non-TCC related causes (median time: 20 months, range: 12–55), compared to 4 (17%) in group 2 (median time: 50, range: 15–60 months).

## Discussion

Traditionally, a nephron-sparing endoscopic approach to UT-TCC was reserved for imperative indications. Recently though, this has been challenged with endoscopy applied more frequently in elective cases. However, as UT-TCC remains relatively uncommon, the literature for endoscopic therapy is accordingly limited. A Cochrane review of the surgical management of UT-TCC in 2011 concluded that there is no high quality evidence available to determine the best surgical management, although current data, albeit limited, suggests a similar oncologic outcome when endoscopy is compared to an open approach [6]. Our study adds to the relatively small number of comparative studies focusing on patients with low grade UT-TCC, and furthers the growing understanding that nephroureterectomy may in fact be overtreatment. In a similar finding to Grasso et al. [7], our results indicate that patients' safety and disease-related mortality are not hindered by a nephron-sparing approach. Whereas there is little risk of disease progression and associated mortality for low-grade UT-TCC, the risk of ureteral and bladder recurrence is significant, and is absolutely dependent on a stringent follow-up protocol. In two separate reports of endoscopic treatment of low-grade UT-TCC with medium to long-term follow-up, upper tract recurrence ranged between 74% and 84%, with cancer-specific survival of 100% [8, 9]. In a report of medium-term follow-up of low and high-grade UT-TCC resected endoscopically, Thompson et al. revealed ureter and bladder recurrence in 55% and 45% respectively, with high-grade and non-Ta staging associated with greater mortality [10]. Similarly, in their review, Cutress et al. identified a pooled recurrence rate of 52% following endoscopic therapy for UT-TCC, with a grade-dependent trend. Further, size (>2 cm), prior history of bladder tumor, and greater than three previous bladder tumor resections were associated with UT recurrence [11]. Our results suggest similar bladder and ureter recurrence rates with excellent disease-related mortality, possibly due to our careful patient selection (tumor size up to 1.5 cm and papillary low-grade appearance at primary resection). As with all other related studies, this study is limited by its retrospective nature as well as relatively long treatment period (ten years), both a result of the rarity of the disease. Nevertheless, it would seem almost impossible to prospectively recruit patients for a randomized study.

## Conclusion

Nephron -sparing endoscopic approach for low grade upper tract TCC in well selected cases is associated with good cancer free progression results and stable renal function and could be considered in patients with normal contra lateral kidney. Patient's compliance is essential due to the rigorous follow-up protocol required.

## Abbreviations

UT-TCC: Upper tract transition cell carcinoma
NUx: Nephro-ureterectomy
eGFR: Estimated glomerular filtration rate
URS: Ureteroscopy
